# Massachusetts Dental Society

**Published:** 1866-11

**Authors:** 


### MASSACHUSETTS DENTAL SOCIETY.

The regular monthly meeting of the Massachusetts Dental Society was held at their rooms, Tremont Temple, last evening, President N. C. Keep, M. D., in the chair, with a full attendance of the members.

The regular Essayist, Dr. I. A. Salmon, read a paper on "Conservative Dentistry." Volunteer papers were read by A. Lawrence, D. D. S., of Lowell, and by Dr. D. G. Harrington, of South Boston.

